# Development of a Dendritic Cell/Tumor Cell Fusion Cell Membrane Nano-Vaccine for the Treatment of Ovarian Cancer

**DOI:** 10.3389/fimmu.2022.828263

**Published:** 2022-02-17

**Authors:** Lei Zhang, Wei Zhao, Jinke Huang, Fangxuan Li, Jindong Sheng, Hualin Song, Ying Chen

**Affiliations:** ^1^ Department of Gynecologic Oncology, Tianjin Medical University Cancer Institute and Hospital, Tianjin, China; ^2^ Key Laboratory of Cancer Prevention and Therapy, Tianjin, China; ^3^ Tianjin’s Clinical Research Center for Cancer, National Clinical Research Centre of Cancer, Tianjin, China; ^4^ Department of Biomedical Sciences and Tung Biomedical Sciences Centre, City University of Hong Kong, Kowloon, Hong Kong SAR, China; ^5^ Department of Anatomy and Histology, School of Basic Medical Sciences, Tianjin Medical University, Tianjin, China; ^6^ Department of Cancer Prevention, Tianjin Medical University Cancer Institute and Hospital, Tianjin, China

**Keywords:** ovarian cancer, dendritic cell, fusion cell membrane, CpG-ODN, cytotoxic T lymphocytes

## Abstract

Ovarian cancer (OC) is a malignant tumor that seriously affects women’s health. In recent years, immunotherapy has shown great potential in tumor treatment. As a major contributor of immunotherapy, dendritic cells (DCs) - based tumor vaccine has been demonstrated to have a positive effect in inducing immune responses in animal experiments. However, the effect of tumor vaccines in clinical trials is not ideal. Therefore, it is urgent to improve the existing tumor vaccines for tumor treatment. Here, we developed a fusion cell membrane (FCM) nano-vaccine FCM-NPs, which is prepared by fusing DCs and OC cells and coating the FCM on the poly (lactic-co-glycolic acid) (PLGA) nanoparticles (NPs) loaded with the immune adjuvant CpG-oligodeoxynucleotide (CpG-ODN). The fusion process promoted the maturation of DCs, thus up-regulating the expression of costimulatory molecule CD80/CD86 and accelerating lymph node homing of DCs. Furthermore, FCM-NPs has both the immunogenicity of tumor cells and the antigen presenting ability of DCs, it can stimulate naive T lymphocytes to produce a large number of tumor-specific cytotoxic CD8^+^ T lymphocytes. FCM-NPs exhibited strong immuno-activating effect both *in vitro* and *in vivo*. By establishing subcutaneous transplanted tumor model, patient-derived xenograft tumor model and abdominal metastatic tumor model, FCM-NPs was proved to have the effect of delaying the growth and inhibiting the metastasis of OC. FCM-NPs is expected to become a new tumor vaccine for the treatment of ovarian cancer.

## Introduction

Ovarian cancer (OC) is one of the most common and fatal malignancies, ranking the third in incidence and the first in mortality among gynecological malignancies. OC affects about 220,000 women worldwide each year (1). Although the traditional therapies combining radiotherapy and chemotherapy with surgery has prolonged the survival of OC patients, the recurrence and even death caused by chemotherapy and radiation resistance have seriously threatened the health and life of women. Therefore, it is crucial to find new treatment strategies (2).

Tumor immunotherapy is a novel therapeutic method, which can not only recognize and kill tumor cells, but also inhibit tumor metastasis and recurrence by activating the patients’ own immune system (3). The currently developed tumor immunotherapies include immune checkpoint inhibitors, Chimeric Antigen Receptor T-Cell (CAR-T) adoptive immunotherapy and tumor vaccines (4, 5). In recent years, tumor vaccines have shown promising therapeutic effect in clinical trials (6, 7). They have the characteristics of high specificity, convenient preparation and relatively low cost. The principal mechanism of tumor vaccines is to induce the activation and proliferation of tumor antigen-specific cytotoxic T lymphocytes (CTL) to kill tumor cells (8). In 2006, the US Food and Drug Administration (FDA) approved the first tumor vaccine in the world, the prophylactic tumor vaccine-Cervarix, which reduces the incidence of human cervical cancer (9). Subsequently, in April 2010, the FDA approved sipuleucel-T (Provenge™) for the treatment of advanced prostate cancer, making it the first autologous active immunotherapy drug and the first truly therapeutic tumor vaccine (10).

It is well known that dual signals are required to initiate immune response of T cells: the MHC I/II molecules (Signal 1) on the antigen presenting cells (APCs) recognize the antigens and present them to T cells, and the co-stimulatory molecules (Signal 2) are involved at the same time (11). Dendritic cells (DCs) are the most powerful and professional APCs (12). Mature DC cells can efficiently capture, process and present tumor antigens to stimulate tumor-specific immune response. There are two types of traditional DC-based tumor vaccines. One is DC vaccines loaded with tumor antigens, including (1) DC vaccines sensitized by tumor antigen peptides, such as DC modified with tumor specific antigen or tumor associated antigen and DC modified with tumor specific epitope peptide (13, 14); (2) DC vaccines sensitized by tumor cell lysates (15); and (3) DC vaccines sensitized by tumor mRNAs (16). The other one is gene-modified DC vaccines, in which the DC cells were transfected with tumor antigen genes, cytokine genes or chemokine genes (17). Although these DC-based tumor vaccines enhance the function of DCs and the anti-tumor immunity of the body, they have various limitations. For instance, gene-modified DC vaccines express low level of antigen and are prone to cause immune tolerance. The DC vaccines sensitized by tumor antigens also failed to achieve the expected effect in clinical applications (18). On the one hand, there are few tumor-specific antigens that have been identified, some tumors even lack of specific tumor antigens. On the other hand, the extracted tumor protein is easily degraded *in vivo*, making the induced immune effect unsustainable to obtain the ideal therapeutic effect.

The fusion of DCs and tumor cells effectively induce anti-tumor effects in the body and has been demonstrated to be an effective vaccine to stimulate the production of tumor-specific CTL (19). DC/tumor fusion cells (FCs) preserve the antigenicity of tumor cells and the function of antigen presentation and T cells activation of DC. FCs can not only continuously and steadily express endogenous tumor antigens, but also express the major histocompatibility complex (MHC)- I and II and the co-stimulatory molecules such as CD80, CD86 and ICAM-1, which are essential for T cell activation (20). The tumor antigens are then continuously presented to CD8^+^ and CD4^+^ T lymphocytes in the form of antigen peptide-MHC (p-MHC) molecular complexes to generate a large number of CTL and memory T cells, which can kill the tumor, reduce the risk of metastasis and prolong the survival time of patients (21). Gong et al. demonstrated that mice inoculated with the FCs of DCs and mouse MC38 cells can induce the production of tumor-specific CTLs *in vivo* (22). Studies have demonstrated that DC/tumor FCs vaccine can induce tumor-specific immune response in a variety of tumors, including melanoma (23), acute myeloid leukemia (24), lung (25), prostate (26), liver (27) and breast cancer (28), in animal models or clinical trials.

However, there are limitations in the application of FCs. The FCs carry the genetic material of tumors that have risks of carcinogenesis. Besides, the preservation and transportation of FCs might be difficult. In order to solve these problems, we extracted the membrane of FCs and combined nanotechnology to prepare FCM-NPs by coating the fusion cell membrane (FCM) on the biodegradable polymer poly(lactic-co-glycolic acid) (PLGA) NPs containing the vaccine adjuvant CpG-oligodeoxynucleotide (CpG-ODN). Nanoparticles provide the platform for antigen and adjuvant loading due to their unique physical (size, morphology, large surface area) and chemical (surface properties, high reactivity) properties (29). The polymer nanomaterial PLGA has been widely used in the field of vaccine adjuvants because of its excellent biocompatibility, low mucosal adhesion toxicity and controllable drug release (30). V. Kroll et al. (31) constructed CpG-CCNPs nano-vaccine with core-shell structure by wrapping CPG-modified PLGA with B16F10 membrane, which promoted the maturation of DCs both *in vitro* and *in vivo*. Mature DCs expressed CD40, CD80, CD86, MHC-II and other antigen presentation signals, secreted IL-6, IL-12 and other cytokines, activated endogenous immune response, and showed good efficacy in the treatment of melanoma. In this study, we demonstrated that FCM-NPs had a good immune-activating effect both *in vitro* and *in vivo*, and had a significant therapeutic effect on allograft, xenograft and metastatic tumor models in mice. In addition, the FCM-NPs was confirmed to have high biosafety. The fused cell membrane nano-vaccine FCM-NPs provides a novel concept for exploring new immunotherapy strategy of OC, and is expected to be applied as a safe and efficient treatment method in clinics.

## Materials and Methods

### Materials

PLGA (50:50, MW 5000) was obtained from Meilune (Dalian, China). CpG ODN was synthesized by Synbio Technologies (Suzhou, China). RPMI-1640, high glucose-DMEM and DMEM/F12 medium, fetal bovine serum (FBS), Penicillin/Streptomycin solution, PBS buffer (pH 7.4), TBST buffer (pH 7.6), BSA, collagenase II, erythrocyte lysis buffer, GM-CSF (from mouse), IL-4 (from mouse), PKH26, CFSE, PEG solution (50% w/v, MW 1450), PMSF, RIPA lysis buffer, PVA and dichloromethane were purchased from Sigma-Aldrich (Darmstadt, Germany). BCA Protein Assay kit, ECL Western Blotting Substrate were purchased from Thermo Scientific (Massachusetts, US). PVDF was purchased from Millipore (Massachusetts, US). SDS-PAGE gel preparation Kit, Coomassie Brilliant Blue Staining and Destaining Reagent, Tris-EDTA buffer (pH 8.0), mouse IFN-γ ELISA Kit, mouse TNF-α ELISA Kit, mouse IL-6 ELISA Kit, 4% paraformaldehyde, Triton X-100, formalin and paraffin were purchased from Solarbio (Beijing, China). Citrate-EDTA Antigen Retrieval Solution, Antifade Mounting Medium (with Hoechst 33342), Hematoxylin and Eosin Staining Kit and MTT Cell Proliferation and Cytotoxicity Assay Kit were purchased from Beyotime (Shanghai, China). Fc Receptor Blocker was obtained from Abace Biotechnology (Beijing, China). Anti- PAX8-PE (ab215953), anti-MHC-II -FITC (ab239229), anti-CD86-APC (ab218757), anti-CD80-PE (ab93507), anti-CD3-FITC (ab24948), anti-CD8-APC (ab237368), anti-CD8-PE (ab272343), anti-CD4-PE (ab252151), anti-Pan cadherin (ab51034, 1:20000), anti-Na^+^/K^+^-ATPase (ab7671, 1:1000), anti-histone H3 (ab1791, 1:2000), anti-COXIV (ab202554, 1:2000), anti-β-actin (ab8226, 1:1000), goat anti-mouse IgG (ab205719, 1:5000) and goat anti-rabbit IgG (ab205718, 1:5000) were obtained from Abcam (Cambridge, UK). Anti-IFN-γ-FITC (505805) and Mouse Treg Flow Kit (FOXP3 Alexa Fluor^®^ 488/CD4 APC/CD25 PE) were purchased from Biolegend (San Diego, CA, US).

### Clinical Specimens

After being reviewed and approved by the ethics committee of Tianjin Medical University Cancer Institute and Hospital, and with the informed consent of patients, the surgically resected tissues of two OC patients were collected and immediately put into the RPMI-1640 medium containing Penicillin/Streptomycin solution. In the biosafety cabinet (BSC-1000IIA2, Sujing, China), the OC tissue was cleaned with PBS for several times, the connective tissues and blood vessels were removed, and the tissues were cut into a size of about 1 mm × 1 mm × 1 mm. Then the tissue block was washed with PBS for 2-3 times, digested with 2% type II collagenase at 37°C for 20 min. The digestion was terminated with the same amount of serum-containing medium. The cell containing solution was centrifuged at 200 g for 3 min, and the supernatant was sucked out and filtered through a 200-mesh filter to collect the single-cell suspension. Then it was centrifuged at 300 g for 5 min, and the cells were resuspended and mixed with the complete medium. The cells were inoculated into the culture flask and cultured in the incubator (MCO-18AC, SANYO, Japan) with 5% CO_2_, constant temperature and humidity. The medium was changed every day to remove the non-adherent cells, and finally the adherent OC cells were obtained. The cells were cultured to 60%-80% confluence for passage.

### Cell Lines

The mouse OC cell line ID8, human normal liver cell line HL7702 and renal tubular epithelial cell line HK-2 were purchased from ATCC. The ID8 cells were cultured in DMEM medium, HL7702 cells were cultured in RPMI-1640 medium and HK-2 cells were cultured in DMEM/F12 medium. All medium was supplemented with 10% FBS and 1% Penicillin/Streptomycin. The culture condition was 37°C and 5% CO_2_.

### Generation of Bone Marrow Dendritic Cells (BMDCs)

The BALB/c mice were purchased form the Laboratory Animal Center of Tianjin Medical University Cancer Institute and Hospital. The method of inducing mouse BMDCs was as described previously (32). Briefly, the femur and tibia of BALB/c mice were taken under sterile conditions, and the bone marrow cavity was rinsed repeatedly with RPMI-1640 medium to collect bone marrow cells. Erythrocyte lysis buffer was added, then the remaining cells were collected by centrifugation and inoculated in a 6-well plate. The medium contained 10 ng/mL GM-CSF and 10 ng/mL IL-4. The cells were cultured for 48 h, the medium was half-changed every two days and the suspended BMDCs cells were collected on day 6. To identify BMDCs, the lysate of ID8 cells was added and co-incubated for 24 h, then flow cytometry was performed to analyze CD86 and MHC-II on BMDCs.

### Preparation of the FCs

BMDCs and OC cells were collected, and the number of cells was adjusted according to BMDC: OC = 2:1. The cells were washed with 0.1% BSA-PBS, centrifugated at 1200 rpm for 5 min, and resuspended with 0.1% BSA-PBS. The OC and BMDC cells were then stained with PKH26 (red) and CFSE (green), respectively. After rinsing with PBS, the cells were centrifuged at 1500 rpm for 5 min, and resuspended in complete medium. After that, the two tubes of cells were mixed into a 50 mL tube and centrifuged at 1500 rpm for 5min. After discarding the supernatant, the cells were placed in a water bath at 40°C for 3 min. Then 300-400 μL of PEG solution preheated at 40°C was slowly added and mixed with the cells thoroughly. The mixture was then water-bathed at 40°C for another 3 min. Afterwards, the preheated PBS was slowly added to terminate the reaction and the cells were centrifuged at 120 rpm for 6 min. The FCs were resuspended with PBS, observed and photographed using a laser scanning confocal microscope (LSCM) (Olympus FV1000, Japan).

### Flow Cytometry Detection of the Molecules on the Surface of FCs

The FCs were harvested and washed with PBS, the concentration was adjusted to 2×10^6^ cells/tube. Then 2 μL of anti- PAX8-PE and anti-MHC-II-FITC was added and incubated at 4°C for 30 min in dark. After rinsing twice with PBS, the supernatant was discarded, and the cells were resuspended with 500 μL PBS for flow cytometry detection and sorting (BD FACSCalibur, US).

### Extraction and Characterization of FCs Membrane

The cell membranes of ID8, DCs and FCs were obtained by hypotonic method and liquid nitrogen repeated freeze-thaw method (33). In brief, the cells were collected and washed twice with pre-cooled PBS, after centrifugation, the cells were resuspended in a hypotonic solution containing 100 μM PMSF. Then the cells were immersed in liquid nitrogen and freeze-thawed repeatedly for 5 times. After returning to liquid state, the solution was centrifugated at 14800 rpm for 10 min, the precipitate was the desired membranes and was named CCM, DCM and FCM, respectively.

As for membrane protein characterization, CCM, DCM and FCM were mixed with loading buffer and heated at 95°C for 5 min to denature the proteins. Then the SDS-PAGE was carried out. After that, the gel was taken out and stained with Coomassie bright blue solution for 1 h, the protein bands were analyzed after overnight decolorization.

### Western Blot

FCs were lysed with lysis buffer and centrifuged at 12000 rpm for 10 min. The supernatant was taken to quantify the protein content with the BCA protein kit (Thermo Fisher, USA). The protein solution (20μg) added with loading buffer was denatured at 95°C and then loaded for SDS-PAGE. After that, the protein on the gel was transferred to the PVDF membrane. Then PVDF was blocked with 5% skim milk for 1 h and then incubated with primary antibodies diluted with TBST at 2-8°C overnight. On the second day, the PVDF was washed with TBST for 3 times, and then incubated with the diluted secondary antibodies at 37°C for 1 h. Finally, the PVDF was developed with ECL developer.

### Preparation and Characterization of FCM-NPs

The water/organic/water (W/O/W) multiple emulsion and solvent evaporation methods were used to prepare the core PLGA-CpG ODN (34). In short, 40 mg PLGA was dissolved in dichloromethane as the organic phase (O), CpG ODN was dissolved in Tris-EDTA buffer as the internal water phase (W), and then the internal W phase was slowly dropped into the O phase under an ultrasonic ice bath to obtain a water-in-oil emulsion. The emulsion was ultrasonically mixed with 2% PVA solution to form W/O/W emulsion, and the magnetic stirring was continued for 2 h. After that, the emulsion was centrifuged (25000 rpm, 30 min) to precipitate the prepared PLGA-CpG ODN (NPs). The NPs were washed with distilled water for three times and then dried in a vacuum drying oven to a constant weight. Next, the same amount of cell membrane and NPs were dissolved in sterile water, magnetically stirred overnight at 4°C. The mixture was extruded through 200 nm polycarbonate membranes for 11 passes using the Avanti mini extruder (US) and centrifuged to remove excess cell membrane debris. The obtained FCM-NPs were freeze-dried and preserved.

For the characterization of FCM-NPs, 1 mg/mL NPs, FCM and FCM-NP were washed with sterile water for three times and dropped on the copper nets with carbon supported film. The samples were observed and recorded using a transmission electron microscope (TEM) (JEM2100, Japan). Then, the ζ potential of NPs, CCM-NPs, DCM-NPs and FCM-NPs was measured using the Malvern laser particle size analyzer (Mastersize 2000, UK).

### Immune Activation of FCM-NPs *In Vitro*

In order to evaluate the activation effect of FCM-NPs on BMDCs *in vitro*, NPs, CCM-NPs, DCM-NPs and FCM-NPs were co-cultured with BMDCs for 48 h. Then the BMDCs were rinsed and incubated with anti-CD86-APC and anti-CD80-PE for 30 min. The fluorescence was analyzed by flow cytometry to evaluate the activation and maturation of BMDCs. Next, in order to assess the level of inflammatory factors secreted by BMDCs stimulated by NPs, CCM-NPs, DCM-NPs and FCM-NPs for 24 h, the content of TNF-α and IL-6 in the cell culture medium of BMDCs were determined using ELISA kits (Beyotime, China). In addition, in order to evaluate whether T lymphocytes could be activated, the above co-cultured BMDCs or FCM-NPs and mouse splenic lymphocytes were co-cultured in a ratio of 1:10 for 48 h. After rinsing, the splenic lymphocytes were incubated with anti-CD3-FITC, anti-CD8-APC and anti-CD4-PE antibodies for 30 min. The fluorescence was analyzed by flow cytometry.

### Immune Activation of FCM-NPs *In Vivo*

All animal experimental protocols were approved by the Experimental Animal Ethics Committee of Tianjin Medical University Cancer Institute and Hospital. First, to assess the retention ability of the vaccine at the injection site, BALB/c mice were injected with equal amounts of CCM-NPs, DCM-NPs, and FCM-NPs at the groin area. At 8 h after injection, the changes of localization and intensity of fluorescence in mice were observed and recorded with a live imaging system (IVIS spectrum, US). The mice were then sacrificed and their spleens and inguinal lymph nodes were removed for fluorescence imaging. To further evaluate the immune response of mice, five groups of BALB/c mice were injected subcutaneously with PBS, NPs, CCM-NPs, DCM-NPs or FCM-NPs. Immunization was performed twice a week for three consecutive weeks (35). On the 4th day after the completion of all the immunization procedures, the whole blood was collected and the mice were sacrificed. The levels of IFN-γ, TNF-α and IL-6 in serum were detected with ELISA kits. In addition, the spleens of mice were collected, cut into small pieces and ground with PBS to obtain tissue homogenate. After washing with PBS for 3 times, the single-cell suspension was obtained by filtering with the 70 μm cell filter. Erythrocyte lysis buffer was added to remove the erythrocyte, and the pure spleen single-cell suspension was prepared after rinsing. The spleen cells were treated with Fc receptor blocker and then incubated with anti-CD3-FITC, anti-CD8-PE and anti-IFN -γ -FITC for 30min. The fluorescence of the cells was analyzed by flow cytometry. For the detection of the proportion of CD4^+^ CD25^+^ Foxp3^+^ regulatory T cells (Treg) in spleen lymphocytes, anti-CD4-APC and anti-CD25-PE were first added to the cell suspension and incubated in dark for 30 min. Then the cells were fixed and permeabilized. After that, the anti-Foxp3-Alexa Fluor 488 was added and reacted in dark for another 30 min. Finally, flow cytometry was conducted for fluorescence detection.

### Therapeutic Effect of FCM-NPs on Subcutaneous Transplantation Tumor

We established an OC subcutaneous tumor model using ID8 cells in 25 female C57BL/6 mice at age 6-8 weeks. The ID8 cells (3×10^6^) were subcutaneously inoculated into each mouse. After three weeks of inoculation, the mice were immunized with PBS, NPs, CCM-NPs, DCM-NPs and FCM-NPs twice a week for three consecutive weeks. The changes of tumor size and body weight of mice were measured every 5 days. After one month of immunization, all mice were sacrificed, the whole blood, tumor tissues, as well as heart, liver, spleen, lung, kidney and other major organs were collected. The liver and kidney function (ALT, AST, BUN, SCr) were analyzed by automatic biochemical analyzer (IDEXX Catalyst One, US). The tumor tissues and major organs were fixed with formalin, then embedded in paraffin and sectioned. The paraffin sections were dewaxed and rehydrated with gradient alcohol, and then subjected to antigen repair and 10% BSA blocking. Next, the sections were incubated with anti-CD3-FITC and anti-CD8-APC at 37°C for 30 min. Finally, the slides were sealed with glycerin containing Hoechst 33342 and examined by a CLSM. In addition, H&E staining was performed on the sections of heart, liver, spleen, lung and kidney.

### Therapeutic Effect of FCM-NPs on Patient-Derived Tumor Xenograft (PDX) Mice

PDX model was constructed in BALB/c nude mice. OC tissues from two clinical patients were xenografted into the left and right groin of the mice, and the tumors grew to a significant size in 1-3 months. Simultaneously, vaccines P^1^DFCM-NPs and P^2^DFCM-NPs were made from OC cells derived from the two patients. Then the tumor-forming mice were immunized twice a week for a total of six times. Changes in tumor size were measured every 4 days. On the 5th day after the end of the immunization, fluorescence in the mice was analyzed with IDEXX Catalyst One to evaluate the selectivity of the vaccines. On the 45th day, all mice were sacrificed, and the tumor tissues were taken out and made into paraffin sections for IF staining with CD3 and CD8 antibodies.

### Therapeutic Effect of FCM-NPs on Metastatic Tumor Model Mice

The C57BL/6 mice aged 6-8 weeks were divided into five groups to construct the model of abdominal metastasis of OC. The mice were immunized with PBS, NPs, CCM-NPs, DCM-NPs and FCM-NPs, twice a week for 6 times in total. After three weeks, each mouse was intraperitoneally injected with 3×10^6^ ID8 cells. One month later, all mice were sacrificed, and the uterine adnexa tissues were taken out for evaluation. In addition, the metastasis of tumor in the abdominal wall was observed, and the number of metastatic tumor nodules were counted.

### Cytotoxicity Test

In order to evaluate the cytotoxicity of FCM-NPs to normal cells, NPs, CCM-NPs, DCM-NPs and FCM-NPs were co-incubated with human normal liver cell line (HL7702) and renal tubular epithelial cells (HK-2) for 24 h, then MTT reagent was added to the medium and reacted for another 4 h. Afterwards, the supernatant medium was discarded, and DMSO solution of 100 μL per well was added. The formazan crystals were completely dissolved by shaking for 10 min. In the end, the absorbance at 570 nm was measured by a microplate spectrophotometer (BioTek Epoch2, US) to evaluate the survival rate of the cells.

### Statistical Analysis

Data analysis was performed using SPSS 22.0 software. Each experiment was performed at least three times in parallel, and all data were exhibited as mean ± standard deviation (SD). Two groups of data were compared and analyzed using the two-tailed student’s *t* test. Differences between multiple groups were analyzed by ANOVA and Boferroni *post hoc* tests. *P* < 0.05 was considered statistically significant.

## Results

### Characterization of FCM-NPs

**Figure 1:**
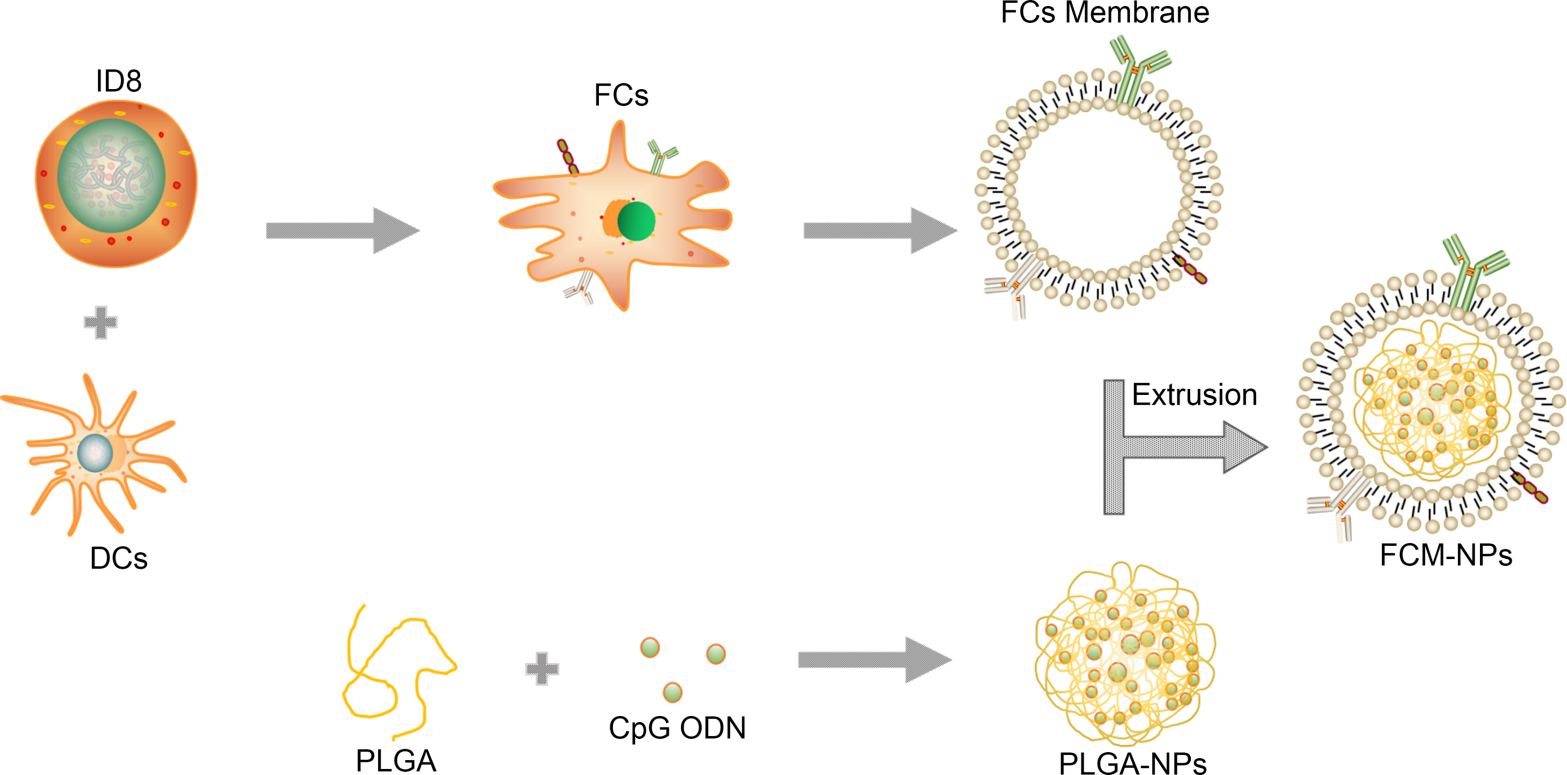
Schematic illustration for the preparation of FCM-NPs. Fusion cells were first prepared from ovarian cancer (OC) cells and mouse dendritic cells (DCs) by PEG method. FCs membrane was then extracted and mixed with CpG ODN-loaded PLGA-NPs, and the FCM-NPs was prepared by extrusion method.

**Figure 2:**
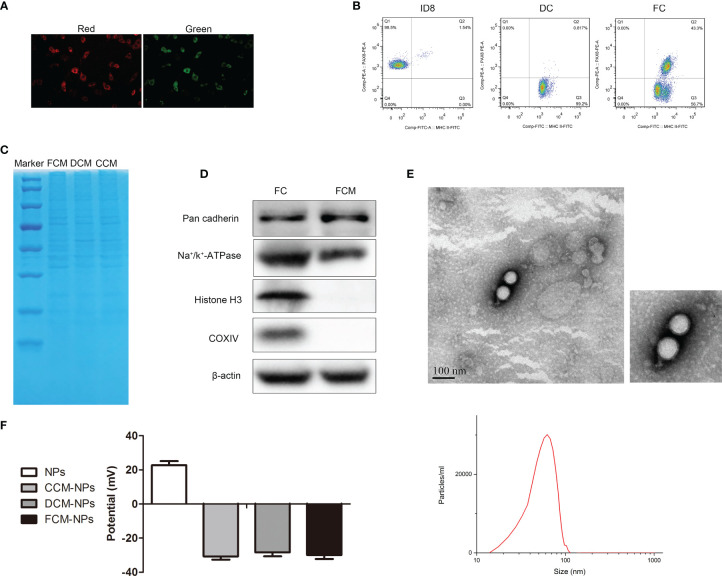
Identification and characterization of FCM-NPs. **(A)** The fusion of DCs and OC cells was observed through CLSM, red fluorescence of PKH26-labelled ID8 cells and green fluorescence of CFSE-labelled DCs. Scale bar = 200 μm. **(B)** Flow cytometry analyses of the CD133-PE on ID8 cells, MHC II-FITC on DCs and the double-labeling on FCs. **(C)** SDS-PAGE analysis of the proteins of ID8 cell membrane (CCM), dendritic cell membrane (DCM) and fusion cell membrane (FCM). **(D)** Western blot analysis of the protein biomarkers of cell membrane, cytoplasm and nucleus in FC and FCM. **(E)** TEM images of FCM-NPs. Scale bar = 100 nm. **(F)** ζ-potential of the NPs, CCM-NPs, DCM-NPs and FCM-NPs.

The procedures of preparation of DC/OC fusion cell membrane nano-vaccine FCM-NPs were shown in **Figure 1**. OC cells were fused with DC cells under the stimulation of PEG. In order to observe the formation of FCs, ID8 and DC cells were labelled with PKH26 (red) and CFSE (green), respectively. As shown in **Figure 2A**, the FCs showed both red and green fluorescence, which confirmed that these two cells were fused under the stimulation of PEG. In addition, we performed flow cytometry to examine the expression of PAX8 and MHC-II on the surface of FCs. PAX8 is the surface biomarker of OC cells (36), and MHC-II is high-expressed on the surface of DCs (18). The proportion of CD133^+^ MHC-II^+^ FCs was 43.3% (**Figure 2B**). Through SDS-PAGE, we found that almost all membrane proteins of OC and DC cells could be found in the FCM (**Figure 2C**), demonstrating the successful construction of FCs. In order to verify that the extracted FCM was pure without nucleus and cytoplasm residues, we detected the expression of several protein biomarkers in FCs by Western blot. The results showed that the membrane biomarkers Pan cadherin and Na+/K+ -ATPase were found in both FC and FCM. However, the protein biomarkers Histone 3 and COXIV that expressed in the nucleus or cytoplasm were not found in FCM (**Figure 2D**). Flow cytometry also showed no nucleic acid residues in FCM (**Figure S1**). Then, the FCM was wrapped on PLGA loaded with the immune adjuvant CpG ODN to obtain FCM-NPs with core-shell structure. It could be seen by TEM that the prepared FCM-NPs appeared as regular spheres with the size of about 70 nm (**Figure 2E**). The ζ-potential of CCM-NPs, DCM-NPs and FCM-NPs were very close, being -31 mV, -29 mV and -30 mV, respectively, while the ζ-potential of the core NPs was 24 mV (**Figure 2F**). These results indicated that we had successfully prepared the nano-vaccines.

### FCM-NPs Activate Immune Cells *In Vitro*

**Figure 3:**
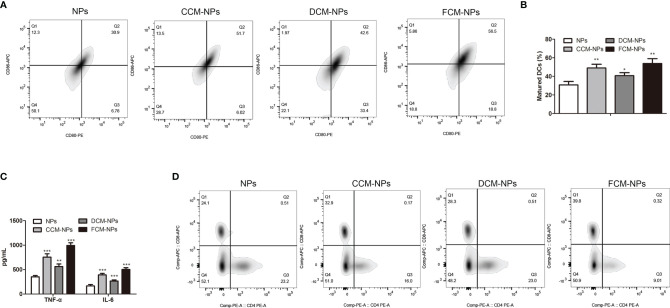
*In vitro* immune activation of FCM-NPs. **(A)** Flow cytometry analyses of the mature biomarkers CD86-APC and CD80-PE of BMDCs co-incubated with NPs, CCM-NPs, DCM-NPs and FCM-NPs. **(B)** Maturation rate of BMDCs in each group. **(C)** Levels of TNF-α and IL-6 secreted by BMDCs determined by ELISA. **(D)** Flow cytometry analyses of the expression of CD8 and CD4 in splenic lymphocytes co-incubated with BMDCs activated by NPs, CCM-NPs, DCM-NPs and FCM-NPs. *P < 0.05, **P < 0.01, ***P < 0.001 *vs* NPs group.

FCM-NPs contain tumor antigens of OC cells, so they can be recognized and phagocytosed by DCs, and induce the maturation of DCs. Mature DCs act as APC and present FCM-NPs to T lymphocytes by up-regulating the expression of MHC-II and co-stimulatory molecules, thus inducing the transformation of T lymphocytes into CTLs. In order to confirm whether FCM-NPs induce the maturation of DCs, we co-cultured NPs, CCM-NPs, DCM-NPs and FCM-NPs with BMDCs for 48 h, and then the expression of co-stimulatory molecules CD80 and CD86 on the surface of BMDCs was detected by flow cytometry. The results showed that the mature BMDCs in FCM-NPs group was the highest (**Figures 3A, B**), indicating that the FCM-NPs were more effective in inducing DCs maturation than CCM-NPs. To avoid fluorescence interference, CpG ODN was deleted in the FCM-NPs used in all *in vitro* experiments, but we found that FCM-NPs carrying CpG ODN were more beneficial to the maturation of DCs (**Figure S3**). In addition, the levels of inflammatory cytokines TNF-α and IL-6 in the supernatant of BMDCs were detected. High level of inflammatory cytokines is an essential condition for the maturation of DCs and an important indicator of the maturation of DCs as well (37). It was found that BMDCs in FCM-NPs group secreted much higher TNF-α and IL-6 than those in CCM-NPs and DCM-NPs groups (**Figure 3C**). After DCs maturation, the captured antigens are processed and presented to T lymphocytes in the form of p-MHC molecular complex, which can be recognized by the T cell receptors and activate the immune function of T lymphocytes (21). Flow cytometry was conducted to detect the proportion of CD8^+^ T lymphocytes in CD4^+^ T lymphocytes after co-incubation of BMDCs treated with different vaccines with mouse spleen lymphocytes. It was found that BMDCs co-cultured with FCM-NPs had the best effect on T lymphocytes activation, the proliferation of CD8^+^ T cell was faster in FCM-NPs group than in CCM-NPs and DCM-NP groups (**Figure 3D**). Furthermore, we investigated whether FCM-NPs could directly activate T lymphocytes. NPs, CCM-NPs, DCM-NPs and FCM-NPs were directly co-cultured with mouse spleen lymphocytes, the results showed that the proportion of CD8^+^ T lymphocytes in FCM-NPs group was higher than that in other groups (**Figure S2**). These results suggest that FCM-NPs activate T lymphocytes through direct and indirect ways to initiate the specific immune response.

### FCM-NPs Activate Immune Response *In Vivo*

**Figure 4:**
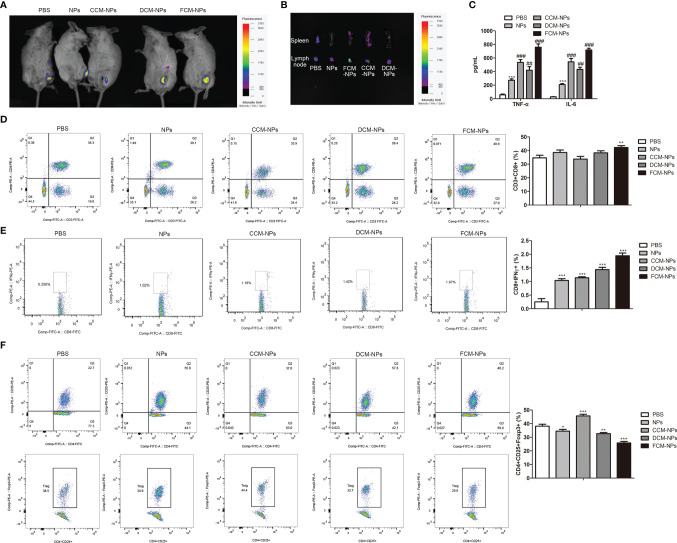
*In vivo* immune activation of FCM-NPs. **(A)** *In vivo* fluorescence imaging of mice at different time points after vaccine injection. **(B)** Fluorescence imaging of spleen and lymph node of each group of mice 36 h after vaccine immunization. **(C)** The secretion of inflammatory cytokines TNF-α and IL-6 in serum detected by ELISA. **P < 0.01, ***P < 0.001 *vs* PBS group, ^##^ *P* < 0.01, ^###^ *P* < 0.001 *vs* NPs group. **(D)** Flow cytometry analyses of the expression of CD3 and CD8 in spleen of mice treated with PBS, NPs, CCM-NPs, DCM-NPs and FCM-NPs. **(E)** Flow cytometry analyses of IFN-γ^+^ and CD8^+^ effector T cell in each group. **(F)** Flow cytometry analyses of the CD4^+^ CD25^+^ Foxp3^+^ Treg cells. *P < 0.05, **P < 0.01, ***P < 0.001 *vs* PBS group.

Encouraged by the results of the *in vitro* experiment, we investigated whether FCM-NPs could activate specific CTL immune response *in vivo*. CpG ODN was included in FCM-NPs used in the *in vivo* experiments. It is well-known that most vaccines are prone to form a local storage of antigen after injection, resulting in slow and sustained release of the antigen to continuously stimulate immune cells to activate a specific immune response (38). In order to explore the retention of vaccines at the injection site, we injected three vaccines CCM-NP, DCM-NP and FCM-NP in the groin region of the mice, and monitored the fluorescence in mice 8 h after injection by IVIS. It is worth noting that the fluorescence intensity in the groin area of mice in the FCM-NPs group was always higher than that in the other two groups (**Figure 4A**), indicating that FCM-NPs have better retention at the injection site. Then the spleens and inguinal lymph nodes of mice were taken out for fluorescence imaging. The fluorescence intensity of inguinal lymph node in the FCM-NPs group was obviously higher than that in the other two groups (**Figure 4B**), indicating that FCM-NPs have strong lymphatic homing ability. In addition, the high levels of inflammatory cytokines TNF-α and IL-6 secreted in serum of the mice in the FCM-NPs group also indicated that FCM-NPs activated the immune response in the mice (**Figure 4C**). Next, we detected the proportion of CD3^+^ CD8^+^ T lymphocytes in mouse splenocytes by flow cytometry. Compared with the PBS group, the proportion of CD3^+^ CD8^+^ T lymphocytes in the FCM-NP group was significantly increased (**Figure 4D**). Similarly, the FCM-NPs stimulated more IFN-γ^+^ CD8^+^ CTLs, which can kill OC cells specially (**Figure 4E**). Besides, FCM-NPs group had a significantly lower proportion of CD4^+^ CD25^+^ Foxp3^+^ Treg cells than the other groups (**Figure 4F**). These results suggest that FCM-NPs can activate tumor-specific immune responses in mice.

### Immune Activation and Therapeutic Effect of FCM-NPs on Subcutaneous Xenograft Tumor Model

**Figure 5:**
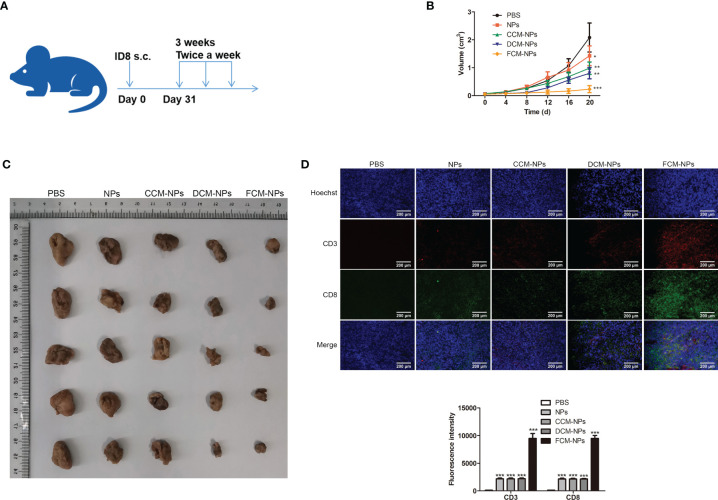
Immune stimulation and therapeutic effect of FCM-NPs on subcutaneous transplanted tumor. **(A)** Schematic diagram of modeling and treatment of subcutaneous transplanted tumor in mice. **(B)** Changes in tumor size of mice in PBS, NPs, CCM-NPs, DCM-NPs and FCM-NPs groups. *P < 0.05, **P < 0.01, ***P < 0.001 *vs* PBS group. **(C)** Images of tumors after the mice were sacrificed. **(D)** Immunofluorescence staining of CD3 and CD8 in tumor sections. Scale bar = 200 μm.

Subcutaneous xenograft tumor model was established by subcutaneous inoculation of ID8 cells to C57BL/6 mice. The establishment of mouse model and the immune process were shown in **Figure 5A**. The mice treated with FCM-NPs had the slowest tumor growth rate over the entire experimental period (**Figure 5B**). After the immunotherapy, the images of the tumors taken from the mice in each group were shown in **Figure 5C**. Tumors in FCM-NPs group were the smallest, while the tumors in PBS, NPs, CCM-NPs and DCM-NPs groups were large, especially in the PBS group. Furthermore, IF staining of CD3 and CD8 in mouse tumor sections showed that the fluorescence intensity of CD3 (green) and CD8 (red) in the FCM-NPs group was the strongest, while the fluorescence intensity of NPs, CCM-NPs and DCM-NPs groups were weaker (**Figure 5D**). These results indicate that FCM-NPs have a strong immune activation effect and good therapeutic effect on subcutaneous xenograft tumor in mice.

### Immune Activation and Therapeutic Effect of FCM-NPs on PDX Model

**Figure 6:**
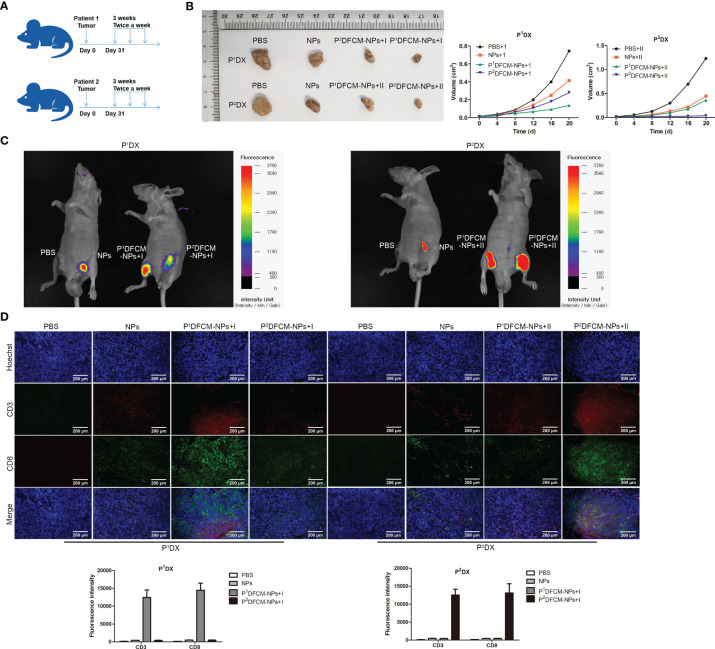
Immune stimulation and therapeutic effect of FCM-NPs on PDX tumor model. **(A)** Schematic diagram of modeling and treatment of PDX tumor in mice. **(B)** The size changes of P^1^DX and P^2^DX tumor in PBS, NPs, CCM-NPs, DCM-NPs and FCM-NPs groups. **(C)** *In vivo* fluorescence detection of the vaccine selectivity in double tumor mode. Scale bar = 200 μm. **(D)** Immunofluorescence staining of CD3 and CD8 in P^1^DX and P^2^DX tumor sections.

OC tissues from two clinical patients were inoculated into the left and right groin of BALB/c nude mice, and each mouse carried two PDX tumors simultaneously. The vaccines P^1^DFCM-NPs and P^2^DFCM-NPs prepared from the OC cells of these two patients were injected into the mice, PBS and NPs were used as control. The procedures of establishment of PDX model and immunotherapy were shown in **Figure 6A**. The changes in the size of tumor P^1^DX and P^2^DX during the treatment period were shown in **Figure 6B** and **Figure S4**. The vaccine P^1^DFCM-NPs had the best inhibitory effect on tumor P^1^DX, and the vaccine P^2^DFCM-NPs had the best inhibitory effect on tumor P^2^DX, indicating that PFCM-NPs have the targeting and therapeutic effect on homologous tumor cells. *In vivo* fluorescence detection was also performed in mice, and the results confirmed the strong selectivity of PFCM-NPs vaccine (**Figure 6D**). After the mice were sacrificed, CD3 and CD8 IF staining was carried out in tumor P^1^DX and P^2^DX sections. The results showed that the fluorescence intensity of CD3 and CD8 was the highest in P^1^DX tumor inoculated with P^1^DFCM-NPs. Similarly, CD3 and CD8 showed the highest fluorescence intensity in P^2^DX tumor inoculated with P^2^DFCM-NPs (**Figure 6C**). The above results indicate that FCM-NPs have strong immune activation and targeted therapeutic effect on homologous PDX tumor cells.

### Therapeutic Effect of FCM-NPs on Metastatic OC Model

**Figure 7:**
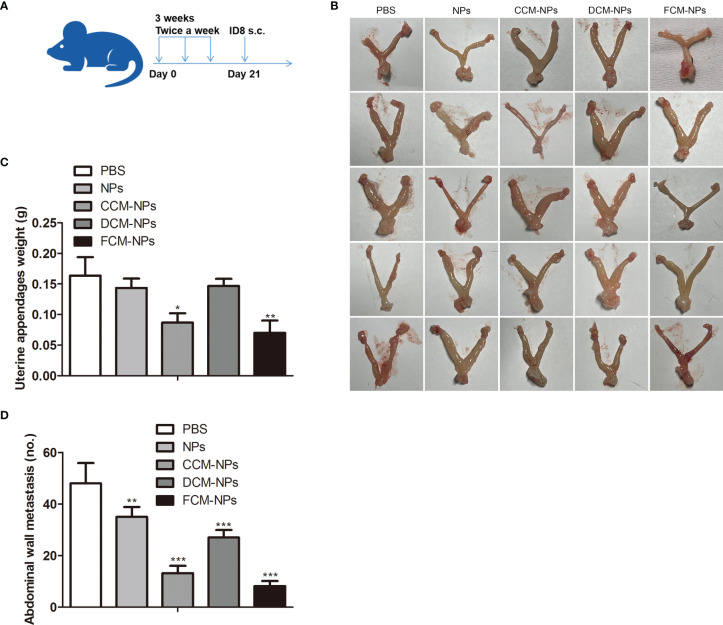
Therapeutic effect of FCM-NPs on abdominal metastatic ovarian cancer model. **(A)** Schematic diagram of modeling and treatment of abdominal metastatic tumor in mice. **(B)** Images of adnexa uteri of mice treated with PBS, NPs, CCM-NPs, DCM-NPs and FCM-NPs. **(C)** Weight of adnexa uteri in mice of each group. *P < 0.05, **P < 0.01 *vs* PBS group. **(D)** The quantitative results of the metastatic tumor nodules on the abdominal wall. **P < 0.01, ***P < 0.001 *vs* PBS group.

To evaluate the efficacy of FCM-NPs in the treatment of metastatic OC, we established an OC abdominal metastasis model and treated mice with vaccines (**Figure 7A**). Mice in PBS, NPs and DCM-NPs groups had large ovarian tumors and obviously increased weight of adnexa uteri, while mice in CCM-NPs group had relatively small ovarian tumor. The adnexa uteri of FCM-NPs group were close to normal and the weight of adnexa uteri was the lowest (**Figures 7B, C**). The trend of abdominal metastasis was basically the same as that of adnexa uteri. We calculated the number of metastatic tumor nodules on the abdominal wall (**Figure 7D**). The number of tumor nodules on the abdominal wall of mice in the PBS group was about six times that of the FCM-NPs group. The number of nodules in CCM-NPs group was less than that of DCM-NPs group, and only FCM-NPs group had the best control effect on abdominal metastasis of OC in mice. These results suggest that FCM-NPs vaccine is beneficial to control the progression and metastasis of OC.

### FCM-NPs Are Safe

**Figure 8:**
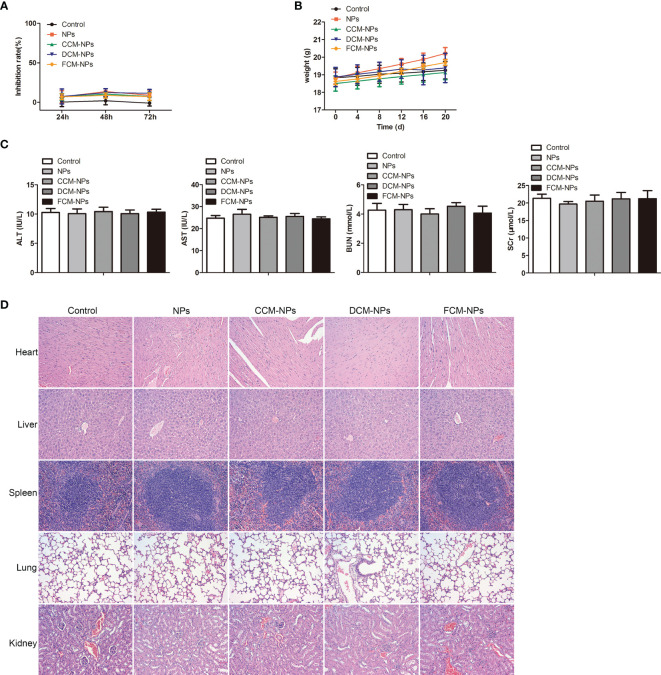
Biosafety evaluation of FCM-NPs. **(A)** The cytotoxicity of FCM-NPs to hepatocyte HL7702 and renal tubular epithelial cell HK-2 was assessed by MTT assay. **(B)** Body weight changes of mice with subcutaneous transplanted tumor and vaccines treatment. **(C)** Liver and kidney function indexes. **(D)** HE staining images of major organs of subcutaneous transplanted tumor model mice after vaccine treatment.

Although our research confirmed that the DC/OC fusion cell membrane nano-vaccine FCM-NPs had a good therapeutic effect on OC and a good control effect on peritoneal metastasis of OC, the safety of FCM-NPs needs to be evaluated before it can be used in clinical practice. We evaluated the effect of FCM-NPs on the cell viability of human normal liver cell HL7702 and renal tubular epithelial cell HK-2 by MTT assay, and the results showed that the inhibition rate of all the five groups was very low and had no significant difference (**Figure 8A**). As for the *in vivo* safety assessment, we monitored the changes in body weight of mice with the subcutaneous tumor treated with the vaccines. The average body weight of mice in FCM-NPs group was always higher than that of the NPs, CCM-NPs and DCM-NPs group, indicating that FCM-NPs have little effect on the normal growth of the mice (**Figure 8B**). Meanwhile, we collected the blood of mice and examined the level of ALT and AST, as well as the BUN and SCr (liver and kidney function indexes). There were basically no differences in the four indexes of the five groups of mice (**Figure 8C**), indicating that the vaccine does not cause liver and kidney damage when used at normal concentrations. In addition, H&E staining was performed on the main organs of the mice in each group: heart, liver, spleen, lung and kidney. H&E staining results in all groups were similar to those in the control group, with no significant difference (**Figure 8D**). The above results confirm that FCM-NPs cause no damage to major organs such as liver and kidney, and have no impact on the growth of the body, so they are safe for clinical trials.

## Discussion

In this study, we developed a fused cell membrane nano-vaccine FCM-NPs for the treatment of OC. FCM-NPs were constructed by fusing the cell membrane of OC and DC cells and coating the fused cell membrane on the surface of PLGA NPs containing vaccine adjuvant CpG ODN. It has been reported that compared with the DC or tumor cells alone and the mixture of both, the fused cells can effectively present endogenous tumor antigen to CD8^+^ T cells, which can be activated into CTL, proliferate and secrete IFN-γ and other cytokines, thus exhibit a specific cytotoxic effect on tumor cells (39). In this study, we found that FCM-NPs activated T cells through direct and indirect ways, these two pathways work together to “amplify” the effect of activating anti-tumor immunity in the host, and effectively kill OC cells. The nano-vaccine NP-FM prepared by Liu et al. (40) using the metal-organic framework carrying the fused cell membrane of mouse breast cancer 4T1 cells and mouse BMDCs was also found to activate T lymphocytes either directly or indirectly through the presentation of DCs.

Although the FCs vaccine has achieved the fusion of cytoplasm and membrane of DC and tumor cells, the nuclei might maintain its independency and integrity. In order to avoid the possible tumorigenicity, in clinical trials, the tumor cells are frequently irradiated with gamma rays before fusion, so that the FCs vaccine applied to the patient will not continue to proliferate in the body to ensure the safety of the vaccine (41). However, there is no uniform standard of radiation dose. Incomplete inactivation may lead to the continued growth of a few cells, while excessive treatment will affect the activity of p-MHC on the surface of FCs, thus reducing the immune activation effect (42). In order to solve this problem and improve the safety of tumor vaccine, we extracted the cell membrane of the FCs as the antigen to activate the immune system, while excluding the cytoplasm and nucleus of FCs.

To further enhance the immunogenicity of the vaccine, we introduced CpG ODN and integrated the antigen-FCM and the adjuvant CpG ODN into the same system with the aid of PLGA NPs. CpG ODN is a synthetic oligodeoxynucleotide containing non-methylated CpG sequence. It can be rapidly internalized by immune cells and bind to TLR9 on the surface of internal vesicles to activate intracellular signaling response (43). CpG ODN quickly activates T and B lymphocytes, promote the production of Th1-type pro-inflammatory cytokines, and directly acts on DCs through the TLR9 signaling pathway to induce the maturation/activation of DCs, which has a broad application prospect in anti-tumor therapy (44). Jie et al. (45) constructed the mucin 1 (MUC1) - maltose binding protein (MBP) anti-tumor vaccine and used CpG ODN1826 as an adjuvant. It was found that the combination of MUC1-MBP-CPG ODN1826 promoted the maturation and activation of DCs. It was also found that MUC1-MBP-CPG ODN1826 significantly prolonged the survival of tumor-bearing mice in *in vivo* experiments. Besides, the Th1 immune response and cell killing effect of CTLs could be induced by MUC1-MBP-CPG ODN1826. In this study, we found that the activation effect of FCM-NPs with CpG ODN on DCs was better than those without CpG ODN, indicating that CpG ODN indeed enhance the immunogenicity of the vaccine.

Currently, there have been studies on the application of FCs vaccine for the treatment OC, but there is a lack of relevant clinical trial data. Koido et al. (46) prepared a FCs vaccine using autologous OC and DC cells from patients. It stimulated the activation of autologous T cells to produce CTLs that against autologous OC cells and secreted cytokines such as IFN-γ. Gong et al. (47) fused OC cells with autologous and allogeneic DCs and obtained similar findings. However, there has no research on the application of FCM nano-vaccines for the prevention and treatment of OC. Here, we confirmed that the nano-vaccine FCM-NPs had a good immune activation effect both *in vitro* and *in vivo*. FCM-NPs induced tumor-specific cell-killing CD8^+^ IFN-γ^+^ T cells, and reduced the number of CD4^+^ CD25^+^ Foxp3^+^ Treg cells simultaneously, promoting the anti-tumor immune response. Moreover, we observed that FCM-NPs had a significant lymph node homing capacity *in vivo*, most likely due to tumor antigens stimulated DCs become mature during the process of fusion. In addition to the up-regulation of MHC and co-stimulatory molecules, DCs also up-regulate the expression of chemokine receptors, that enhance the migratory ability of FCM-NPs from peripheral non-lymphoid tissues to secondary lymphoid tissues such as draining lymph nodes and spleen (48).

In addition to demonstrating the strong immune-stimulating effect of FCM-NPs, we also conducted a systematic evaluation on the efficacy of FCM-NPs against OC. Subcutaneous xenograft tumor model, PDX tumor model and abdominal metastasis tumor model was constructed to evaluate the therapeutic effect of the vaccine. In the subcutaneous xenograft tumor model, we observed that FCM-NPs prepared from mouse ID8 cells had a good inhibitory effect on subcutaneous OC cells. However, in clinical applications, the selection of tumor cells is one of the factors that impact the effect of vaccine. Individualized treatment with patients’ own tumor cells may produce better results (49). In order to better manifest the clinical foresight of the experimental results, we constructed a double-tumor PDX model with OC cells from two patients, and treated them with PDFCM-NPs prepared from OC cells of these two patients. It was found that PDFCM-NPs had stronger immune activation and therapeutic efficacy on homologous OC. This result indicates that the FCM nano-vaccine has homologous targeting effect. Rao et al. (50) also found that the tumor cell membrane vaccines have the characteristic of targeting specificity in the tumor vaccine prepared by coating NPs with head and neck squamous cell carcinoma membrane. Therefore, using autologous tumor cells of patients to construct FCM-NPs vaccine will achieve ideal therapeutic effect in clinical application.

One limitation of this study is that the DCs used in the construction of FCM-NPs were DCs derived from mouse bone marrow, and whether the use of patients’ own DCs or healthy human DCs for cell fusion had an impact on the therapeutic effect was not evaluated. Although it has been reported that there is no significant difference in the CTL induction ability between tumor cells fused with allogeneic DCs and autologous DCs (47), the therapeutic effect of FCM-NPs vaccine constructed from homologous and allogeneic DCs by PDX model warrants further evaluation, and which will be the direction of our future study. In addition, because advanced OC is prone to metastasis to the fallopian tube, uterus, and abdominal organs (51), whether FCM-NPs could inhibit the metastatic OC is also the key question. Cheng et al. (35) used DC membrane to encapsulate PLGA NPs loaded with IL-2 to prepare a “mini DC” nano-vaccine for *in vivo* treatment of OC and found that the “mini-DC” vaccine showed a superior effect in inhibiting the metastasis of OC. In this study, we conducted experiments on the abdominal cavity metastasis mouse model, and confirmed that FCM-NPs had a significant inhibitory effect on the metastasis of OC in adnexa uteri and abdominal wall. With the help of these three types of mouse OC models, we finally confirmed the excellent inhibitory effect of FCM-NPs on the growth and metastasis of OC *in vivo*.

In addition, we have confirmed that FCM-NPs are safe, and they have no undesirable effect on liver and kidney function or cause damage to major organs when used in the therapeutic concentration range. However, more rigorous and meticulous *in vivo* animal studies are needed to ensure the safety and effectiveness of the vaccine before conducting clinical trials. Furthermore, as a tumor vaccine, it should combine the function of prevention and treatment, but this research focuses on the therapeutic effect of nano-vaccine FCM-NPs, for the prevention function, it was only studied in the metastatic tumor model, the preventive effect of FCM-NPs also needs be studied in depth in other models.

In conclusion, the DC/OC FCM-NPs nano-vaccine developed in this research is expected to be a new generation of tumor vaccine for effective treatment of OC.

## Data Availability Statement

The original contributions presented in the study are included in the article/**Supplementary Material**. Further inquiries can be directed to the corresponding author.

## Ethics Statement

The studies involving human participants were reviewed and approved by the ethics committee of Tianjin Medical University Cancer Institute and Hospital. The patients/participants provided their written informed consent to participate in this study. The animal study was reviewed and approved by the Experimental Animal Ethics Committee of Tianjin Medical University Cancer Institute and Hospital.

## Author Contributions

LZ and YC drafted the manuscript. LZ, YC, and WZ conceived and designed the study. LZ, WZ, FL, and JS performed the acquisition of data. HS, FL, and JS performed the analysis and interpretation of data. LZ, YC, and FL obtained funding. All authors read and approved the final manuscript.

## Funding

This work was supported by Tianjin Science and Technology Plan (20YFZCSY00040) and Tianjin Key Medical Discipline(Specialty) construction Project.

## Conflict of Interest

The authors declare that the research was conducted in the absence of any commercial or financial relationships that could be construed as a potential conflict of interest.

## Publisher’s Note

All claims expressed in this article are solely those of the authors and do not necessarily represent those of their affiliated organizations, or those of the publisher, the editors and the reviewers. Any product that may be evaluated in this article, or claim that may be made by its manufacturer, is not guaranteed or endorsed by the publisher.

## Supplementary Material

Supplementary Figure 1 Flow cytometry analyses of the nucleic acid in FC and FCM. Flow cytometry analyses of the Hoechst 33342 stained FC and FCM.

Supplementary Figure 2 Flow cytometry analyses of the activation of T lymphocytes. The expression of CD8 and CD4 of T lymphocytes incubated with NPs, CCM@NPs, DCM@NPs and FCM@NPs for 48 h was determined by flow cytometry.

Supplementary Figure 3 The activation effect on DCs of FCM@NPs with CpG is better than that without CpG. **(A)** DCs maturation rates after the stimulation of with and without CPG-carrying vaccines. **(B)** Secretion of TNF-α and IL-6 of DCs treated with different vaccines.

Supplementary Figure 4 Changes of tumor size in PDX tumor model mice. Changes of the tumor size of P^1^DX and P^2^DX in PBS, NPs, P^1^DFCM and P^2^DFCM groups.
