# Supplementary figures and images for: Development of a Dendritic Cell/Tumor Cell Fusion Cell Membrane Nano-Vaccine for the Treatment of Ovarian Cancer
Source: Front Immunol. 2022 Feb 17;13:828263. doi: 10.3389/fimmu.2022.828263 (PMC8893350; doi:10.3389/fimmu.2022.828263)

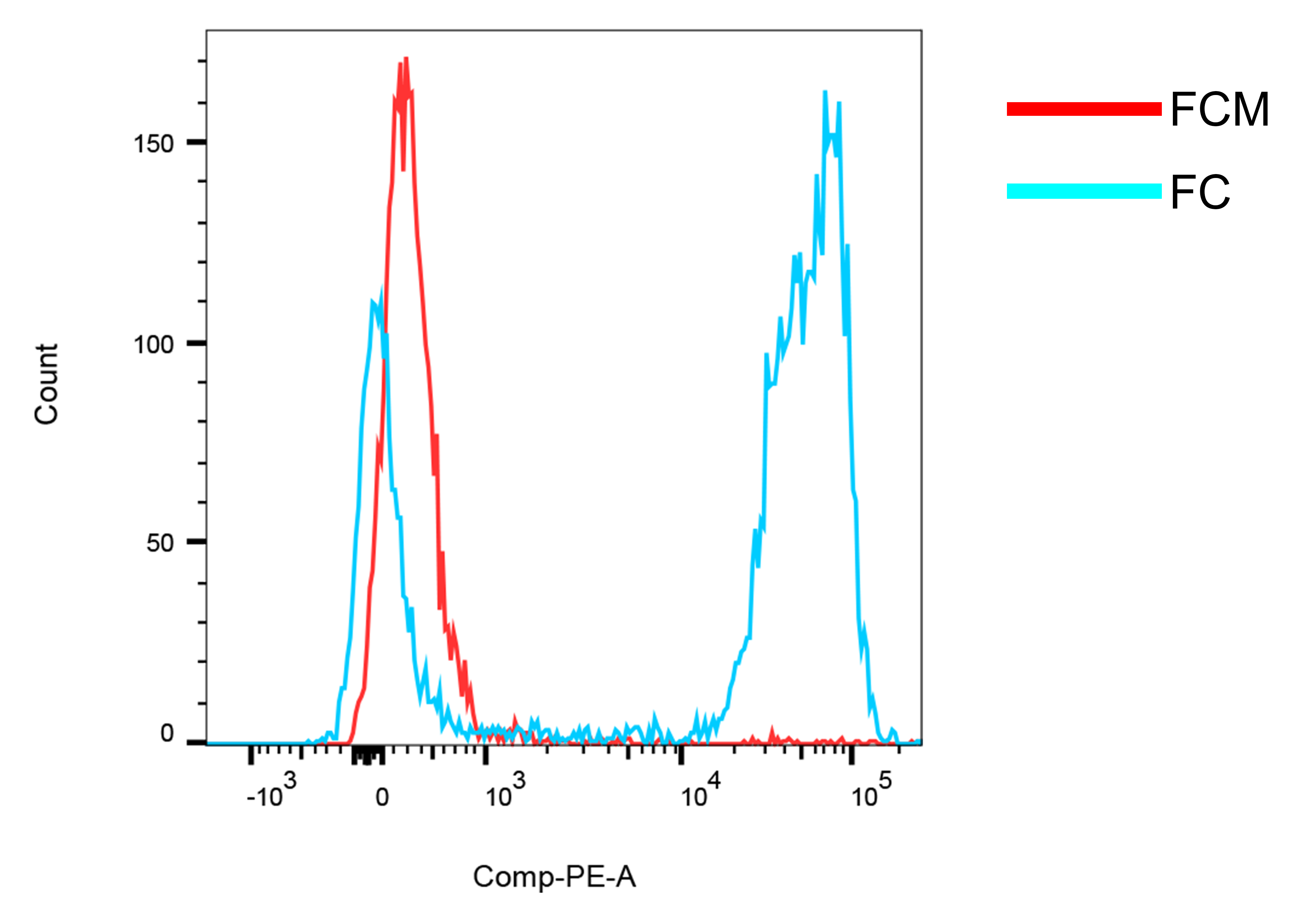

Supplement: Supplementary Figure 1 — Flow cytometry analyses of the nucleic acid in FC and FCM. Flow cytometry analyses of the Hoechst 33342 stained FC and FCM. [file Image_1.tif]

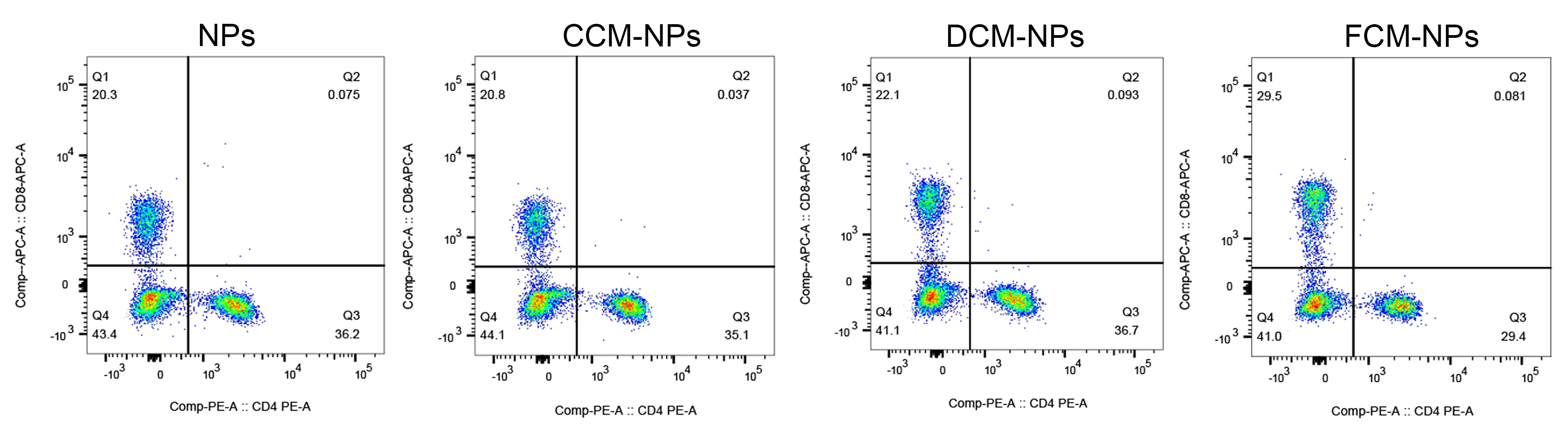

Supplement: Supplementary Figure 2 — Flow cytometry analyses of the activation of T lymphocytes. The expression of CD8 and CD4 of T lymphocytes incubated with NPs, CCM@NPs, DCM@NPs and FCM@NPs for 48 h was determined by flow cytometry. [file Image_2.tif]

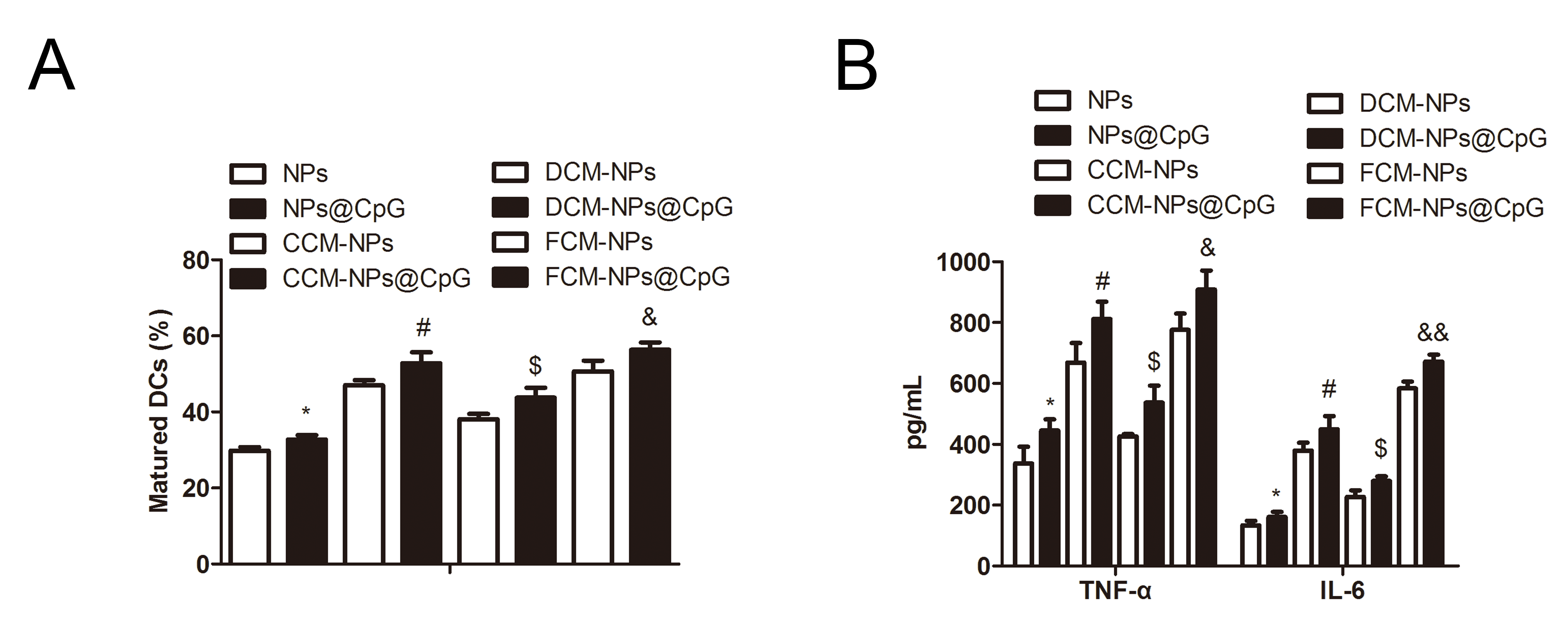

Supplement: Supplementary Figure 3 — The activation effect on DCs of FCM@NPs with CpG is better than that without CpG. (A) DCs maturation rates after the stimulation of with and without CPG-carrying vaccines. (B) Secretion of TNF-α and IL-6 of DCs treated with different vaccines. [file Image_3.tif]

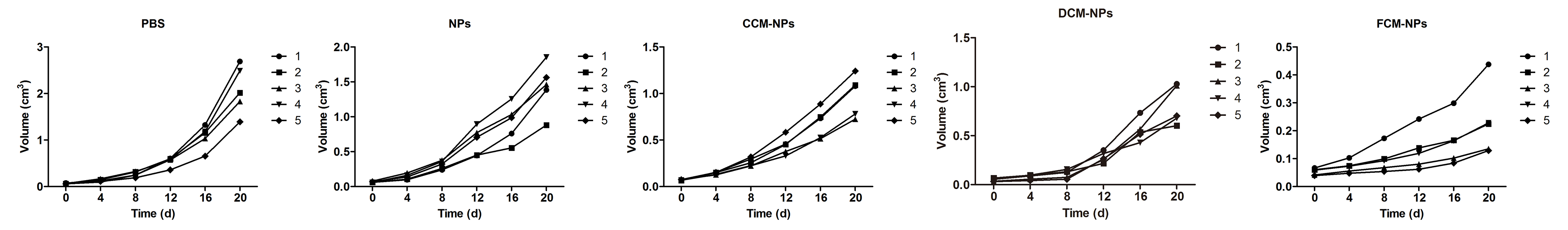

Supplement: Supplementary Figure 4 — Changes of tumor size in PDX tumor model mice. Changes of the tumor size of P1DX and P2DX in PBS, NPs, P1DFCM and P2DFCM groups. [file Image_4.tif]
